# Current and prospective therapeutic strategies: tackling *Candida albicans* and *Streptococcus mutans* cross-kingdom biofilm

**DOI:** 10.3389/fcimb.2023.1106231

**Published:** 2023-05-11

**Authors:** Yijun Li, Shan Huang, Jingyun Du, Minjing Wu, Xiaojing Huang

**Affiliations:** ^1^ Fujian Key Laboratory of Oral Diseases and Fujian Provincial Engineering Research Center of Oral Biomaterial and Stomatological Key Lab of Fujian College and University, School and Hospital of Stomatology, Fujian Medical University, Fuzhou, China; ^2^ Department of Endodontics, Stomatological Hospital of Xiamen Medical College, Xiamen, China; ^3^ Stomatological Hospital, Southern Medical University, Guangzhou, China

**Keywords:** biofilm, *Candida albicans*, *Streptococcus mutans*, cross-kingdom interaction, therapeutic strategies

## Abstract

*Candida albicans* (*C. albicans*) is the most frequent strain associated with cross-kingdom infections in the oral cavity. Clinical evidence shows the co-existence of *Streptococcus mutans* (*S. mutans*) and *C. albicans* in the carious lesions especially in children with early childhood caries (ECC) and demonstrates the close interaction between them. During the interaction, both *S. mutans* and *C. albicans* have evolved a complex network of regulatory mechanisms to boost cariogenic virulence and modulate tolerance upon stress changes in the external environment. The intricate relationship and unpredictable consequences pose great therapeutic challenges in clinics, which indicate the demand for *de novo* emergence of potential antimicrobial therapy with multi-targets or combinatorial therapies. In this article, we present an overview of the clinical significance, and cooperative network of the cross-kingdom interaction between *S. mutans* and *C. albicans.* Furthermore, we also summarize the current strategies for targeting cross-kingdom biofilm.

## Introduction

Biofilms are highly organized communities of microorganisms attached to biotic and abiotic surfaces and surrounded by a protective extracellular matrix. The human oral cavity harbors nearly 700 species of microorganisms that live as biofilms (Valm, 2019). Generally, the composition, activities, and interactions among these microbial communities maintain normal levels of fluctuation. Once endogenous dysbiosis of the oral microbial communities occurs due to some driven factors, these biofilms could transform into a pathogenic state to trigger infectious diseases such as caries, periodontitis and peri-implantitis and so on (Lamont et al., 2018; Sedghi et al., 2021). These driven factors include excessive carbohydrate consumption, reduced salivary flow, alcohol or tobacco overuse, poor oral hygiene, and so on (Sampaio-Maia et al., 2016; Rosier et al., 2018). In the past years, reports from governments and global health authorities have stated the seriousness of caries which occur in all ages especially in children and older adults (Gibney et al., 2021; Hugo et al., 2021). Dental caries, one of the most common diseases in the world, has been associated with a result of collective activities of dental biofilm. It refers to a process that cariogenic species metabolize external sugars and produce acids, thereby resulting in a lower pH local environment and the demineralization of the dental hard tissue over time.

Earlier studies have attributed bacterial biofilms not fungal biofilms to the main pathogenic factors of caries. Though the involvement of fungi like *Candida* species in oral mucosa infections is well recognized, the research about fungus in caries is poorly investigated owing to their small isolated populations from the human mouth (Nikawa et al., 1998; Maijala et al., 2007). In recent years, the role of *Candida* species infection in the etiology of caries has gained more attention. Some *Candida* species especially *Candida albicans* (*C. albicans*) is involved in the development of caries. *C. albicans* is a commensal fungus that colonizes the skin, oral mucosa, vaginal mucosa, and other anatomical parts. The virulence of *C. albicans* lies in its ability to form dense biofilms that comprise yeast cells, pseudo hyphae, and hyphae within the abundant matrix (Nobile and Johnson, 2015).

The cariogenic potential of *C. albicans* has been demonstrated in several parts. Firstly, *C. albicans* can not only adhere to hydroxyapatite-like substrates but also form co-adhesion with some pioneer bacteria to achieve tight adhesion to tooth surface (Montelongo-Jauregui and Lopez-Ribot, 2018; Thanh Nguyen et al., 2021). Secondly, even though *C. albicans* could not highly decompose sucrose due to the lack of α-glucosidase, it can still metabolize fructose, glucose, or lactose to produce short-chain carboxylic acids to lower the pH of the surrounding environment, thereby promoting the demineralization of hard tissue (Klinke et al., 2009). Thirdly, *C. albicans* shows peculiarities in terms of acid tolerance, which is related to the proton pump and H^+^-ATPases on the cell membrane surface (Bowman and Bowman, 1986). It was proposed that the acid-producing and acid tolerance ability of *C. albicans* is superior to *Streptococcus mutans* (*S. mutans*) in some extreme conditions like lower pH environment. Fourthly, the most important cariogenic feature of *C. albicans* is its interspecies networks with different bacteria, whose consequences are associated with elevated virulence and more severe infection (Du et al., 2021; Fourie et al., 2021). The presence of *C. albicans* would undoubtedly alter the ecological niche shared with oral bacteria, which may encourage interspecies cooperation for the benefit of each other. For this review, we sought to update the readers concerning the clinical evidence, interaction relationship, and antimicrobial strategies advancements of cross-kingdom biofilms consisting of *C. albicans* and *S. mutans*.

## Clinical evidence of the co-existence of *S. mutans* and *C. albicans*

Early childhood caries (ECC) is perhaps the most common form of dental caries that disproportionately affects millions of underprivileged preschool children worldwide. An earlier investigation found a higher amount of *C. albicans* in ECC children when compared to caries-free children, though the role of *C. albicans* in caries etiology has not been established yet at that time (de Carvalho et al., 2006). A clinical study has compared the microbial composition of caries plaque samples from 30 ECC children versus plaque samples from caries-free children and found a higher detection rate of *C. albicans* in ECC plaque sites (Srivastava et al., 2012). A number of clinical studies also supported the positive correlation between *C. albicans* carriage and ECC severity (Nascimento et al., 2016; Xiao et al., 2018b). Besides, two research teams pointed out that the genotypic distribution of *C. albicans* is also associated with the caries experience of children (Raja et al., 2010; Qiu et al., 2015). In addition to *C. albicans*, a high proportion of *S. mutans* was also detected in the plaque where *C. albicans* was co-isolated in ECC lesions (Bachtiar and Bachtiar, 2018; Sridhar et al., 2020). A previous study has reported that the total isolation frequency of *S. mutans* and *C. albicans* from plaque and saliva of ECC children was 66% and 18% respectively (Fragkou et al., 2016). Lately, a cohort study used 16s rRNA amplicon sequencing to identify the microbiota of saliva and supragingival plaque from severe early child caries (S-ECC) children (Xiao et al., 2018a). In comparison with data obtained from caries-free children, their results suggested that the presence of *C. albicans* in S-ECC children is responsible for bacterial composition change, which is characterized by an increased abundance of highly acidogenic and aciduric microbiota like *S. mutans*. In addition, as one of the virulence traits in S-ECC, the plaque glucosyltransferase (Gtf) enzymatic activity was significantly higher with the increased abundance of *C. albicans*. In addition to the above observations, it was found that the carriage of *C. albicans* and *S. mutans* in the infant oral cavity displayed positive associations with the mother’s *C. albicans* carriage in a study of 101 mother-infant pairs (Ramadugu et al., 2020). Their results suggested the infant microbiota may have a prenatal origin. Based on the findings establishing the association of *S. mutans* and *C. albicans* in caries initiation and progression, a 2022 study of cross-sectional study wanted to investigate the association of *S. mutans* and *C. albicans* with ECC recurrence (Garcia et al., 2021). Taking a large sample from 143 children who were caries-free, treated for ECC with no recurrence, or treated for ECC and experiencing recurrence within 6 months, the study showed co-infection with *C. albicans* and *S. mutans* was strongly associated with caries recurrence.

Root caries is another subtype of severe caries except ECC, which mainly occurs in middle-aged and elderly people. *Streptococcus*, *Lactobacillus*, and *Actinomyces* species are recognized as the prominent pathogenic microorganisms in root caries. *C. albicans* was initially identified in root carious dentin (Fure, 2004), however, researchers did not find the reasons for its preference in colonizing the root surface. Another study reported higher counts of *C. albicans* in the root caries lesions of middle-aged and old-aged patients with an incidence range from 27% to 31% (Zaremba et al., 2006), implying *C. albicans*’s colonization and the resulting acidification activities and activation of dentin tubule collagen dissolve may play essential roles in root caries. The co-existence and interaction of *S. mutans* and *C. albicans* in root caries were observed in recent researches via imaging and sequencing techniques. A recent clinical study of dental plaque composition analysis obtained from older Chinese people revealed that versus those without root caries, patients with root caries are abundant in *C. albicans* carriage and also positively correlated with increased *S. mutans* numbers (Du et al., 2020).

The above-mentioned clinical studies demonstrated the positive occurrence and correlation of *C. albicans* and *S. mutans* in the dental plaque of ECC and root caries. It can be inferred that their interaction contributes to the pathogenesis of caries.

## Increased understanding of the complex cross-kingdom interactions of *C. albicans* and *S. mutans*

**Figure 1:**
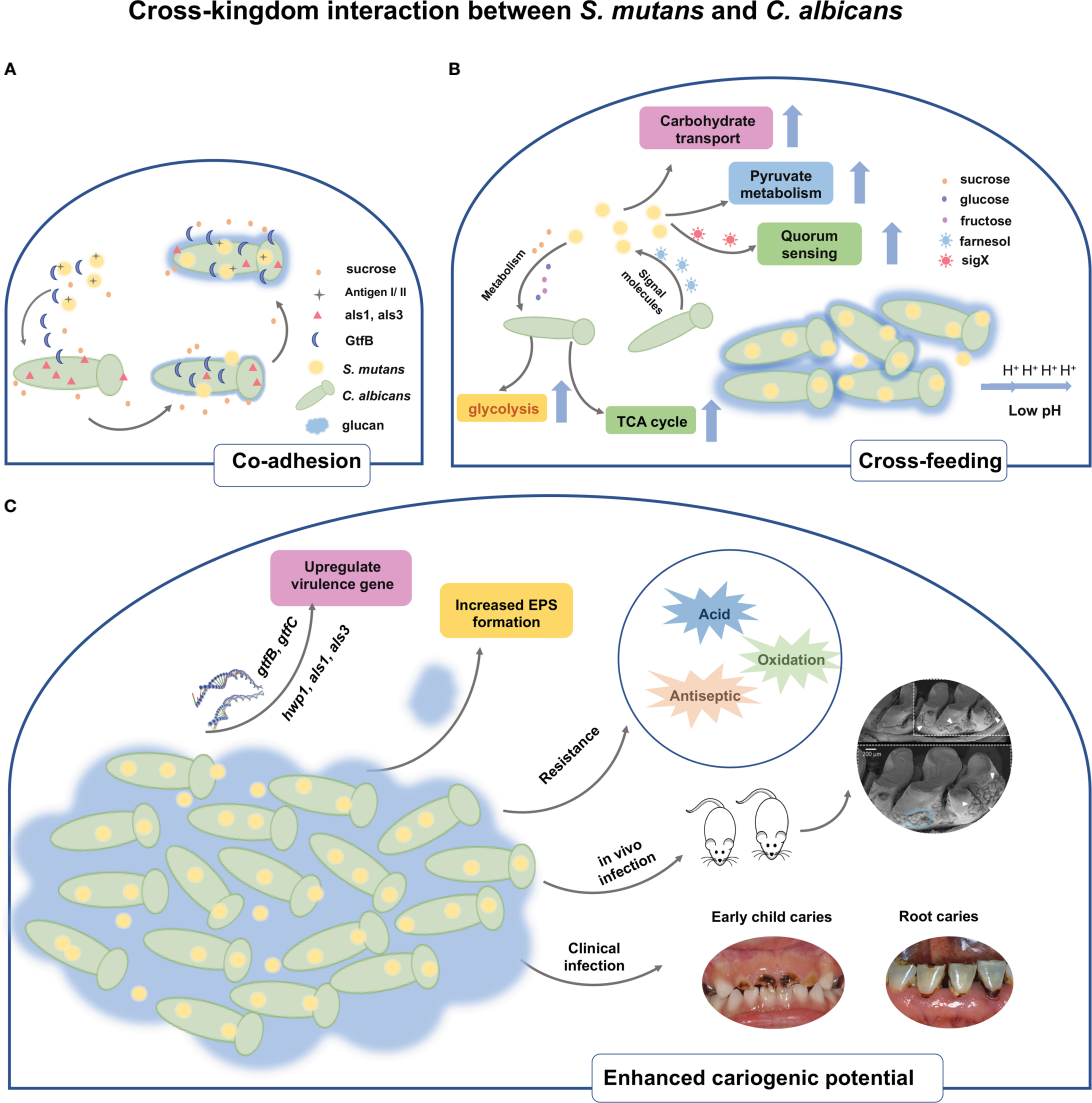
Schematic representation of the cross-kingdom interaction between *S. mutans* and *C. albicans*. **(A)** The physical interaction of *C. albicans* and *S. mutans* is mediated through antigenI/II and als adhesins. Under sucrose environment, *S. mutans*-derived GtfB binds to *C. albicans* cell surface, thereby producing glucans on the *C albicans* cell surfaces and in turn allowing *S. mutans* cells to adhere to the glucans. **(B)** In addition to physical interaction, cross-feeding metabolism occurs between *S. mutans* and *C. albicans*. *S. mutans* relies on sucrose as a nutrient source and metabolize sucrose into fructose and glucose, both of which are nutrient supply of *C. albicans*. Meanwhile, *S. mutans* significantly increases the biochemical activities of such as sugar transport systems, glycolysis, pyruvate degradation, and TCA cycle. Conversely, the presence of *C. albicans* contributes to carbohydrate transport and metabolic/catabolic process of *S. mutans*. The quorum sensing molecule of *C. albicans*, farnesol, could enhance cell growth, microcolony development, and Gtf activity of *S. mutans*. **(C)** Upregulated expressions of virulence factors both in *S. mutans* and *C. albicans* are observed when co-cultivating together. The interaction also stimulates the EPS matrix production and biofilm biomass formation. Owing to the increased virulence factors, the cross-kingdom biofilm of *S. mutans* and *C. albicans* displays resistance towards acid, oxidative, and antiseptic stress. Co-infection of *S. mutans* and *C. albicans in vivo* substantially increase biofilm accumulation, hard tissue demineralization, and carious lesions in rats. Clinical evidence demonstrated that the cross-kingdom relationship of *C. albicans* and *S. mutans* contributes to the progression of ECC and root caries.

Pathogens in multi-species biofilms may act synergistically, antagonistically, and competitively during colonization. The co-infection of *C. albicans* and other bacteria could influence the expression of virulence traits, the human immune system response and antimicrobial resistance with consequences of increased morbidity, and health problems (Cavalcanti et al., 2015; Bernard et al., 2020). Considering the severities of co-infection due to the interaction of *C. albicans* and *S. mutans*, scientists have thoroughly investigated the interaction mechanisms in order to search for novel therapeutic solutions. Their interaction mechanisms lie in different stages of biofilm formation (**Figure 1**).

## Physical interactions

The physical interaction between microorganisms is the prerequisite of coexistence, which promote co-adhesion and proximity, further contributing to chemical interactions and biofilm maturity. Scientists have attempted to reveal the exact mechanisms behind the interaction of *C. albicans* and oral bacteria in past decades (Jenkinson et al., 1990; Cavalcanti et al., 2017; Zhou et al., 2022). The agglutinin-like sequence family containing eight members is located in the cell wall of *C. albicans* and is the key to *C. albicans* in the interaction with other bacteria during biofilm development (Peters et al., 2012; von Ranke et al., 2018). For instance, evidence has shown the cell-to-cell contact between oral streptococci and *C. albicans* is mediated by bacterial surface adhesion SspA/SspB and hyphal cell wall ALS3 adhesins of *C. albicans* (Silverman et al., 2010). This suggests *S. mutans*, belonging to oral streptococci, may possess the same physical contact mode with *C. albicans* as *Streptococcus gordonii* (*S. gordonii*) and *Streptococcus oralis*. Indeed, antigen I/II of *S. mutans* mediates the interaction between *S. mutans* and *C. albicans*. Loss of *spaP* gene coding for antigen I/II could lead to a significant reduction of *C. albicans* numbers in the dual-species biofilms both *in vitro* and *in vivo* (Yang et al., 2018). The required adhesin proteins in mediating the attachment to surfaces or cells of *C. albicans*, such as als1 and als3, are downstream targets of Efg1. The recent study has used *C. albicans* homozygous knockout strains including *ΔΔefg1* and *ΔΔals1/ΔΔals3* to investigate the role of adhesins in the interkingdom colonization between *S. mutan*s and *C. albicans* (Ren et al., 2022). Notably, the *ΔΔefg1* and *ΔΔals1/ΔΔals3* deletion led to a severe reduction of coassembly between *C. albicans* and *S. mutans*. The findings indicated that *C. albicans* could directly bind to *S. mutans* through these surface proteins.

However, it seems that the co-adhesion between *C. albicans* and *S. mutans* is not just dependent on cell-cell physical direct binding, but is also mediated by Gtf enzymes secreted by *S. mutans*. Scanning electron microscope observation represented the co-adhesion of *C. albicans* and *S. mutans* within a dense matrix in the sucrose environment (Pereira-Cenci et al., 2008). Based on this finding, it has been hypothesized that the glucosyltransferase enzyme of *S. mutans* may contribute to the interaction of *C. albicans* and *S. mutans* during co-cultivation. Then Gregoire et al. explored the co-adhesion behaviors of *C. albicans* and *S. mutans* (Gregoire et al., 2011). The authors found that glucosyltransferase B, produced by *S. mutans*, binds tightly to the surface of *C. albicans* yeast cells and contributes to the *in situ* production of glucan by *C. albicans* cells. Interestingly, the *in situ*-formed glucans can provide adhesive sites for *S. mutans* binding, as well as enable the tight contact of *C. albicans* and *S. mutans*, and concomitantly enhance the attachment of *C. albicans* to the hydroxyapatite (HA) surface. The latter of the same research group used *gtfB* knock-out *S. mutans* strains, defective in regulating the formation of exopolysaccharides, to culture with *C. albicans* (Falsetta et al., 2014). The confocal laser scanning microscope (CLSM) image showed that the co-species biofilm of *△gtfB S. mutans* and *C. albicans* presented fairly small, random and sparse clusters, whose attachment force is too weak and mechanically easy to remove from HA disks. To further understand the exact locations of GtfB binding to *C. albicans* cell surface, another study has investigated the precise surface molecule of *C. albicans* when binding to GtfB (Hwang et al., 2017). *C. albicans* mutants with defects in genes encoding mannoprotein biosynthesis, including *och1* and *pmt4*, showed reduced abilities in binding with GtfB and robust biofilm formation. Atomic force microscopy (AFM) is an advanced tool for imaging and unveiling biophysical properties of the binding interactions between microorganisms. By using this technology, the binding force and dynamics of GtfB–*C. albicans* has been measured (Hwang et al., 2015). The data showed the bond between GtfB with *C. albicans* is highly stable with a low dissociation rate. Besides, the binding strength of GtfB to the *C. albicans* surface was ~2.5-fold higher and the binding stability was substantially higher (~20-fold), as compared with the GtfB adhesion to *S. mutans*. Another study also applied this method to evaluate the presence/absence of glucan on the binding forces of *S. mutans* to *C. albicans* (Wan et al., 2021). As a result, the glucan coating on *C. albicans* dramatically enhanced the binding between these two microorganisms. Notably, the binding force of *S. mutans* to glucan-coated *C. albicans* was substantially higher (~6-fold) than the one from *S. gordonii*–*C. albicans*. This result indicated the higher binding force of *S. mutans*-*C. albicans* would help *S. mutans* boost competitiveness against *S. gordonii* under some cariogenic conditions.

Previous and current findings reveal *C. albicans* adhesins, *S. mutans*-derived GtfB and *C. albicans*-produced glucans are critical for the co-adhesion and subsequent co-species biofilm development. In addition, the strong binding force of *S. mutans* and *C. albicans* may account for, at least in part, the competitiveness of cross-kingdom biofilms. It is noteworthy that these findings are constructed in high sucrose conditions. However, the human saliva composition varies not comprising sucrose alone, therefore, further investigations of the different saliva components on the *S. mutans* and *C. albicans* interaction are required to gain more knowledge of the pathogenesis of cross-kingdom biofilms.

## Metabolic interaction

After the initial adhesion, the cells start to proliferate to form microcolonies, forming the basal layer of the biofilm. Different research groups have discovered that *S. mutans* and *C. albicans* stimulate each other’s growth when co-cultivation with sucrose environment. Paradoxically, *C. albicans* is not capable of utilizing sucrose effectively (Williamson et al., 1993). It seems that complex signals, cross-feeding, and metabolic interactions between *S. mutans* and *C. albicans* influence the proliferation rate, growth pattern, and virulence of co-species biofilm. This was experimentally demonstrated by Wu et al., who observed that cell membrane vesicles of *S. mutans* decompose sucrose into glucose and fructose to offer nutrient supply for *C. albicans* from their Benedict’s and Seliwanoff’s tests (Wu et al., 2020). *C. albicans* grows at a much higher rate when supplemented with glucose or fructose than sucrose. For *S. mutans*, it has been shown that conditioned medium from *S. mutans* - C*. albicans* biofilm favors its growth, Gtf activity and microcolony development (Kim et al., 2017). In addition, some matrix components like α-Mannan and β-1,3-Glucan purified from *C. albicans* stimulate *S. mutans* adherence and biofilm formation (Khoury et al., 2020). These results implied that *S. mutans* and *C. albicans* could share metabolites and cooperatively metabolize complex molecules to complement the metabolic requirements for biosynthetic pathways. The latter two studies provide some evidence accounting for the complex mechanisms responsible for enhanced growth in *S. mutans* and *C. albicans* dual-species biofilm from the gene aspect. The first finding is proposed by Ellepola et al., showing *S. mutans* enhanced *C. albicans* gene expression related to carbohydrate metabolism, including sugar transport systems, glycolysis, pyruvate degradation to ethanol and acetate production, the tricarboxylic acid cycle, and the electron transport chain (Ellepola et al., 2019). Furthermore, the transcriptomic changes also somewhat influence the protein level, some proteins associated with carbohydrate metabolism of *C. albicans* were also significantly increased in mixed-species biofilms. Carbohydrate metabolism is a crucial part of dental caries development. Another research studied how *C. albicans* influences the transcriptome of *S. mutans* in an established co-cultivation biofilm model (He et al., 2017). Their transcriptome data suggested that significant difference in gene expression of *S. mutans* between single and dual-species biofilm. Gene ontology function analysis showed the genes of *S. mutans* is related to carbohydrate transport and metabolic/catabolic process were regulated. For instance, lac operon is responsible for lactose transport and metabolism, encoding the tagatose 6-phosphate pathway (Zeng and Burne, 2021). Conversely, the accelerated metabolism of lactose can also make *C. albicans* transform from yeast to virulent hyphae. Other small chemical molecules, including farnesol, competence-stimulating peptide (CSP) and *sigX*, are also involved in the mediation of the growth kinetics between *C. albicans* and *S. mutans* (Jarosz et al., 2009; Sztajer et al., 2014). Farnesol is a lipophilic molecule that accumulates in cell membranes and mediates quorum sensing system of *C. albicans.* A low level of farnesol (25-50 μM) could boost *S. mutans* growth and stimulate *gtfB* expression, which is associated with the microcolony development of *S. mutans* (Kim et al., 2017). Though a higher level of farnesol inhibited *S. mutans* growth, interestingly, the presence of *S. mutans* appeared to reduce the farnesol production of *C. albicans. SigX* is an alternative sigma factor for genes that control the uptake of exogenous DNA in *S. mutans*, whose expression can be activated by the CSP and *sigX*-inducing peptide (XIP). Sztajer et al. demonstrated the induction of *sigX* and high activation of the complete quorum sensing regulon of *S. mutans* when in co-culture with *C. albicans* (Sztajer et al., 2014). These findings have evidenced that quorum sensing pathways of these two species participate in the cross-kingdom interaction between *S. mutans* and *C. albicans*. However, the exact mechanisms underlying how these molecules modulate competition and cooperation in cross-kingdom biofilms remain unclear, but their potential significance is high.

## Impact on virulence traits

As soon as cells attach to the surface and grow, the biofilm continues to grow by a formation of extracellular polymeric substance (EPS) until it reaches the maturation phase. The EPS matrix, one of the biofilm virulence factors, contributes to intercellular interactions and biofilm stability (Flemming and Wingender, 2010). Mixed *C. albicans*-*S. mutans* biofilms display an abundance in EPS matrix compared to their monospecies biofilm format. The increased EPS matrix is conducive to nutrients retention, which in turn facilitates cross-feeding between them. However, very little is known about the exact compositions in the extracellular matrix of *S. mutans* and *C. albicans* cross-kingdom biofilms. Further work should be conducted to define the structure and functions of EPS matrix components in the cross-kingdom biofilm. Despite this, numerous genes associated with EPS matrix have been investigated, with the redundancy of these genes further complicating the structure of the biofilm. Multiple authors have ascribed the increase in EPS matrix to the upregulated *gtfB e*xpression in the *S. mutans*-*C. albicans* cross-kingdom biofilm. GtfB is responsible for the synthesis of water-insoluble glucose, which is linked by α-1,3 glucosidic linkages to form glucan, an essential fraction of the EPS matrix (Senadheera et al., 2007). In addition to *gtfB*, *S. mutans* also enhances the expression of *C. albicans hwp1*, *als1*, and *als3* in cross-kingdom biofilms (Ellepola et al., 2017). These upregulated genes are critical for the adherence, filamentous growth, and biofilm formation of *C. albicans* (Nobile et al., 2006; Lombardi et al., 2019).

*S. mutans* and *C. albicans* cross-kingdom biofilms are potent in acidogenicity owing to their metabolic mutualism relationship that facilitates the exchange of carbohydrates between partners. Sampaio et al. have assessed the acidogenicity of *S. mutans* and *C. albicans* cross-kingdom biofilm by measuring pH of culture medium and found the pH drops to an extreme value of 4.5 (Sampaio et al., 2019), collaborating with the findings of Kim (Kim et al., 2020). This low pH condition made *S. mutans* and *C. albicans* be dominant species since this is a true “acid shock” for other oral commensals as they would cease growth or not survive below 5.5. Moreover, *ldh* gene expression related to acid production increased while *ureC* and *arcA* expression associated with acid counteraction decreased (Du et al., 2020). The distinct features of acidogenicity in cross-kingdom biofilm could result in more differential damage to mineralized tooth tissue. In addition to acidogenicity, the progression of caries is intertwined intimately with acid tolerance by caries pathogens. It is conceivable that EPS matrix in cross-kingdom biofilm would act as a physical and protective barrier for microorganisms. Several recent studies have also investigated the genetic regulation of acid tolerance in *S. mutans* and *C. albicans* co-species biofilm. For instance, the gene *PHR2*, which encodes putative glycosidases required for proper cross-linking of β-1,3-glucans and β-1,6-glucans *C. albicans*, was significantly upregulated in cross-kingdom biofilm (Matsushika et al., 2016). Another upregulated gene is *fabM*, which is responsible for monounsaturated fatty acids synthesis and is necessary for *S. mutans* survival in an acidic environment (Fozo and Quivey, 2004).

The cross-kingdom interaction may aid *C. albicans* or *S. mutans* to survive under some extreme conditions. It is well documented that the biological niches in which oral microorganisms reside are rich in physiological and molecular cues involving low pH, high expression of some specific enzymes, high concentration of hydrogen peroxide (H_2_O_2_), and so on. To mimic this situation, Lobo and co-workers have compared the survival rate of *C. albicans* or *S. mutans* dual-species biofilm with their corresponding single-species biofilm after exposure to different stress conditions (Lobo et al., 2019). They found that both *C. albicans* and *S. mutans* in dual-species biofilm displayed higher survival rate than single biofilm when responding to 3% H_2_O_2_ and 0.2% chlorhexidine (CHX), implying the dual-species biofilm has a higher tolerance ability. In addition, the increased resistance of *C. albicans* towards fluconazole was also observed when in co-culture with *S. mutans.* Kim et al. found that the *S. mutans*-derived EPS matrix, which is dramatically increased during co-existence, coats the fungal cell, thus hindering the uptake of fluconazole (Kim et al., 2018). For the phenomenon of the increased tolerance to disinfectants in *C. albicans*-bacterial cross-kingdom biofilm, several recent reviews have stated the possible mechanisms involved in interspecies protection (Nogueira et al., 2019; Yan et al., 2022). Regarding *S. mutans*-*C. albicans* cross-kingdom biofilm, the increased matrix proven before is responsible for the tolerance of the whole populations within the biofilm. As known, the disinfectants or antimicrobials may encounter limited penetration difficulties through the matrix and hardly reach the deepest layers of the biofilm in their active form. While the alterations like persister cell differentiation, upregulation of drug efflux pumps, or genetic resistance during *C. albicans* and *S. mutans* co-cultivation have not been explored.

## Therapeutic strategies

**Figure 2:**
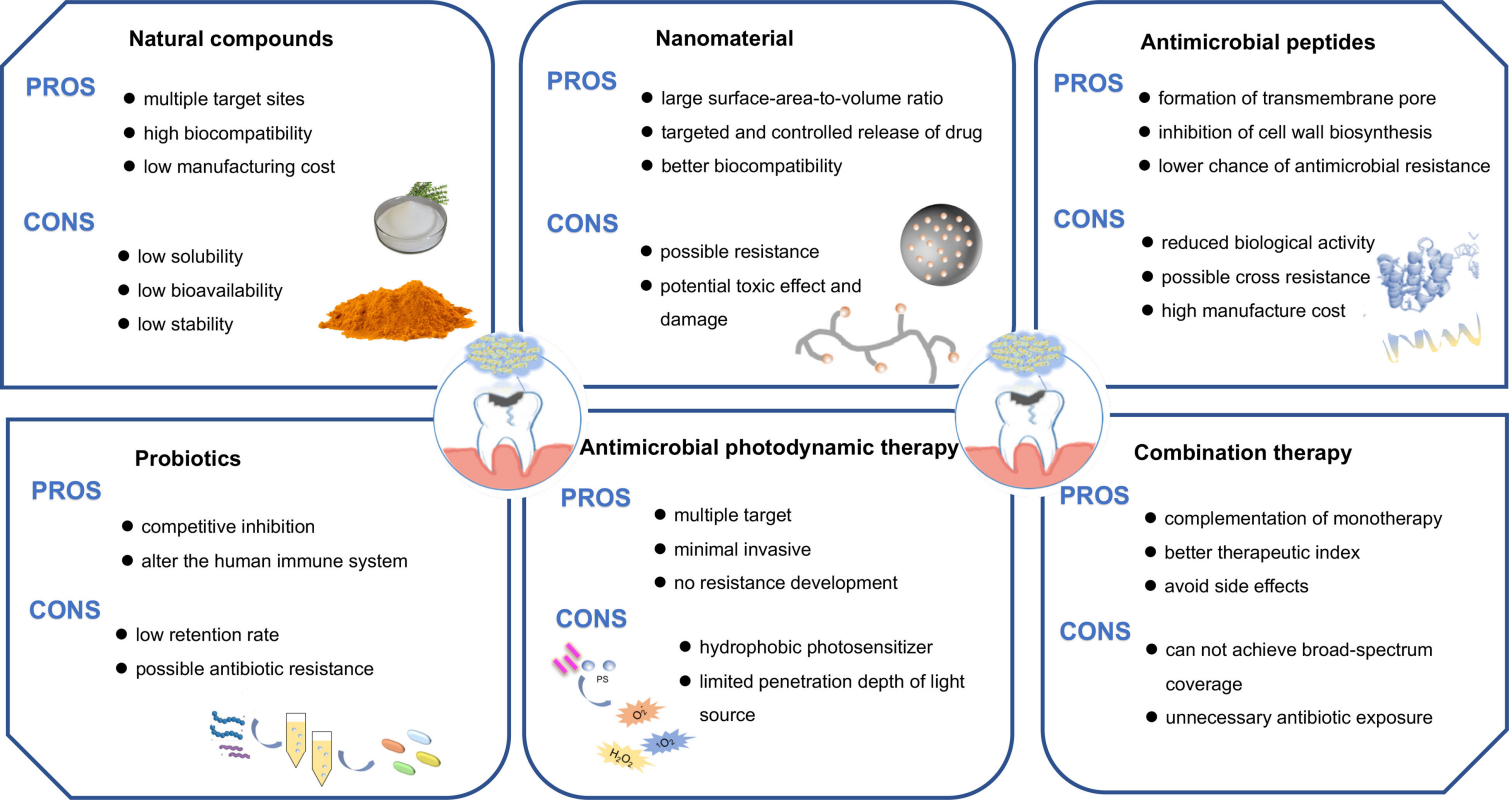
Different approaches for the treatment of the cross-kingdom biofilm. Biofilms could be inhibited by employing natural compounds, nanomedicine, antimicrobial peptides, antimicrobial photodynamic therapy, probiotics or combination therapy.

Clinical and experimental evidence demonstrated that the cross-kingdom relationship of *C. albicans* and *S. mutans* contributes to the progression of ECC and root caries. The rising incidence of antimicrobial tolerance, as well as the involvement of the notorious cross-kingdom biofilms in caries, has raised an urgent need in the search for novel therapeutic strategies. The traditional treatments of carious lesions are done with mechanical removal by burs, hand excavators, or other techniques. Alternatively, Er: YAG laser ablation and fluorescence-aided caries excavation are also introduced in carious dentin removal following the notion of minimally invasive techniques. However, no matter how carefully done, healthy parts of the tooth will be removed, even causing pulp exposure and pulp complications. On account of the hardness of deciduous teeth and root cementum being low, the occurrence rate of pulp exposure increased. Additionally, *C. albicans* and *S. mutans* are still left behind in the dentine after excavation, which contributes to caries recurrence. In recent years, attempts have been made to treat cross-kingdom biofilms by utilizing natural products, developing new nanomaterials or applying laser therapy (**Figure 2**).

## Natural compounds

Natural compounds, mainly phytochemicals, could be used as an alternative for complementing antibiotics in the treatment of multiple disorders (Mishra et al., 2020). A number of studies have been published where natural compounds were tested for antimicrobial efficacy against oral biofilms (Karygianni et al., 2015). The results have ascertained the role of these natural products in preventing microorganism adhesion, inhibiting the EPS matrix formation, and thus reducing the deleterious effects of the pathogenic biofilms. Several antimicrobial mechanisms of natural compounds are also identified, including inhibition of cell division, block of efflux pump activities, disruption of cell membrane, and quench of quorum sensing.

The most common type of natural compound in oral application is phenolics. For example, curcumin is a natural polyphenol extracted from the rhizomes of Curcuma longa, which has been extensively investigated due to its wide range of pharmacological properties (Zheng et al., 2020). Curcumin has been proven to reduce biofilm biomass and inhibit the EPS matrix formation of *C. albicans* and *S. mutans* cross-kingdom biofilm (Li et al., 2019). Besides, curcumin remarkably downregulated the expression of *gtfB* and *gtfC* in *S. mutans*, two essential genes for meditating the cross-kingdom interaction between *C. albicans* and *S. mutans*. Another finding of suppressing the expression levels of some quorum sensing genes was also observed in this study, which is consistent with the former studies on the functions of phytochemicals. Thymol, a phenol monoterpene compound, is a promising agent for the prevention of biofilms formed by gram-negative and gram-positive microbes. A recent study has investigated the effects of thymol on *C. albicans* and *S. mutans* dual-species biofilm (Priya et al., 2021). Their data revealed that 300 μg/mL thymol exhibits anti-biofilm activity in *C. albicans* and *S. mutans*, both single-species and dual-species biofilms. The antibacterial mechanism against *C. albicans* or *S. mutans* is through the inhibition of several physiological activities, including yeast-hyphal transformation, filamentation, acidogenicity and acidurity. In the larvae infection model, the treatment with thymol led to reduced CFU counts of the infection site and significantly improved the survival of larvae. In addition, other types of natural compounds belonging to terpenoids or essential oils have been tested for their anti-biofilm capacity on the cross-kingdom biofilms. Caffeic acid phenethyl ester (CAPE), a classical type of terpenoids that extracts from propolis, exerts its potential in suppressing the growth, biofilm formation and EPS synthesis of *C. albicans* and *S. mutans* (Yin et al., 2022). The efficacy of CAPE is probably due to its down-regulation action in the expression level of *gtf* genes. Similarly, eugenol as a major component of essential oil, has been shown to inhibit the formation of *C. albicans* and *S. mutans* dual-species biofilm by reducing the viability of microorganisms and disrupting the biofilm structure. A few natural compounds with the potential of anti-biofilm activity are summarized in **Table 1**.

**Table 1:** Summary of selected studies investigating antimicrobial effects of natural compounds on cross-kingdom biofilms.

Name	concentration	biofilm growth/substrate	Treatment time	Microbial number reduction	Biofilm biomass reduction	Cytotoxicity	*In vivo* model	Proposed mechanisms	Reference
Curcumin	0.5 mM	48h/96-well plate	24 h	*Sm*: 69% *Ca*: 38%	24%	NM	NM	Inhibition of EPS formation and virulence factors	(Li et al., 2019)
Thymol	300 μg/mL	24h/ glass surface	24 h	*Sm*: 3.5 log *Ca*:2.5 log	50%	NM	larve infection	Inhibition of yeast-to-hyphal transition, hyphal-to-yeast transition, filamentation, and acidogenicity and acidurity of dual-species biofilms	(Priya et al., 2021)
CAPE	20, 40, 80μg/mL	24 h/96-well plate	24 h	Reduced (No specific number)	Reduced (No specific number)	Human oral keratinocytes	NM	Inhibition of adhesion, EPS synthesis and cariogenic gene expression	(Yin et al., 2022)
GLE	0-7 mg/mL	24 h/96-well plate	24 h	NM	51 % (7 mg/mL)	NM	NM	Inhibition of glucan formationof *S. m*	(Campbell et al., 2020)
GSE	0-7 mg/mL	24 h/96-well plate	24 h	NM	70 % (7 mg/mL)	NM	NM	Inhibition of glucan formationof *S. m*	(Campbell et al., 2020)
PPFGT	0.0.1-0.63 mg/mL	48 h/96-well plate	48h	NM	Reduced (No specific number)	NM	NM	Inhibition of growth and EPS	(Farkash et al., 2019)
Eugenol	50,100,200 μg/mL	48 h/glass coverslips	48 h	*Sm*:1.01 log (200 μg/mL) *Ca*:1.46 log (200 μg/mL)	NM	NM	NM	Disruption of cell membrane and matrix structure	(Jafri et al., 2019)
Tyrosol	50,100,200 mM	48 h/acrylic resin, hydroxyapatite	48 h	*Sm*:4 log (200 mM) *Ca*: 5log (200 mM)	NM	NM	NM	NM	(Arias et al., 2016)

*Sm*, *Streptococcus mutans*; *Ca*, *Candida albicans*; GSE, gesho stem extract; GLE, gesho leaf extract; NM, Not mentioned; EPS, Extracellular Polymeric Substance; ROS, Reactive oxygen species; PPFGT, polyphenon from green tea.

The wide exploration of the effects of these natural compounds is due to their unique characteristics like multiple target sites, high biocompatibility, low possibility of resistance and manufacturing cost. However, some drawbacks of natural extracts such as low solubility in water, low stability during storage, low bioavailability, and ingestion efficiency, hinder the full development of their use. One feasible method of improving the efficient delivery of natural compounds is to use nanotechnology in terms of increased bioavailability, targeting, and controlled release. The encapsulation of natural compounds by lipid-based nanoparticles is proven to be an efficient strategy to overcome those limitations. Additionally, the above-mentioned studies lack cytotoxicity evaluation, comparison with the current antimicrobials, and *in vivo* infection models to validate the drug efficacy. Thus, further studies should be conducted in this regard to test the actual effectiveness of these natural compounds before entering into clinical application.

## Nanomaterials

Nanomaterials, with dimensions generally in the 1–100 nm range, are versatile and bioactive and have received much attention in biomedical applications. They offer numerous advantages over conventional drugs, such as large surface-area-to-volume ratio, ultra-small sizes, and excellent chemical and physical properties (Benoit et al., 2019; Rubey and Brenner, 2021). Due to the intrinsic antimicrobial potential, organic and inorganic nanomaterials could act as biofilm-targeting agents or drug carriers. The antimicrobial mechanisms of nanomaterials on various microorganisms are not realized entirely. It is known that nanomaterials would penetrate microbial cell membrane easily and inhibit cell respiration and other essential biological processes. Additionally, nanomaterials could induce the burst of oxidative stress in microorganisms, leading to microbial death.

Chitosan is an excellent antimicrobial agent, a nontoxic polysaccharide, and nontoxic to various types of human cells. They show efficacy against *in vitro* and *in vivo* bacterial infections of a wide range of microbial species. Chitosan nanoparticles have been demonstrated to decrease the viability and the biofilm biomass of *C. albicans* and *S. mutans* biofilm after 18 h incubation (Ikono et al., 2019). However, treatment with chitosan nanoparticles for 3 hours did not affect the established dual-species biofilm. The results implied nanoparticles alone might have a limited effect on the cross-kingdom biofilms, which was consistent with the previous studies (Ikono et al., 2019; Aati et al., 2022). Therefore, scientists have worked on another way to take advantage of nano-sized materials. They could be engineered as drug carriers to transport promising drugs into the infection site, with the end goal of making the drug more efficient. Thanks to the encapsulation of the nanocarriers, the drug is protected from these threats and then taken up by the target cells to ensure the therapeutic effect. A representative example is the encapsulation of phloroglucinol into chitosan nanoparticles (CSNPs) via the ionic gelification method (Khan et al., 2022). Chitosan nanoparticles have been extensively employed to deliver different agents. The chitosan loaded with phloroglucinol (PG-CSNPs) exhibited inhibitory effects on the cross-kingdom biofilm of *C. albicans* and other bacteria in a concentration-dependent manner. Additionally, PG-CSNPs could enhance the effectiveness of several antibiotics in the mature cross-kingdom biofilms. CHX is regarded as a gold standard antimicrobial owing to its broad antimicrobial spectrum and excellent antimicrobial effectiveness. However, the higher concentration of CHX is toxic to the surrounding cells and likely to develop resistance. In order to solve this problem, iron oxide magnetic nanoparticles (IONPs) and chitosan (CS) have been synthesized, which protect CHX from rapid degradation and reduce the dose required for therapeutic success (Vieira et al., 2019). The result also revealed that IONP-CS-CHX could effectively reduce the biofilm biomass of *C. albicans and S. mutans* dual-species biofilm. Moreover, INOP-CS-CHX showed a similar or superior effect in reducing viable microbial amounts and EPS matrix compared to free drugs. Both inorganic and organic nanoparticles can be modified to release the encapsulated drug under different stimuli, such as light, pH, and heat. Ito et al. formulated the multifunctional nanocarrier with a pH-responsive element, which achieved pH-triggered “smart release”, to exert selective antibiofilm activity by targeting the specific pathogenic microenvironments (Ito et al., 2022). They studied the effects of the pH-responsive nanoparticles loaded with saturated farnesol (NPC-Far) on antibiofilm activity and teeth enamel demineralization. The NPC-Far could prevent local demineralization of tooth enamel by inhibiting biofilm formation on enamel. **Table 2** summarizes the nanomaterials with inhibitory effects against cross-kingdom formed by *C. albicans* and *S. mutans*.

**Table 2:** Summary of selected studies investigating antimicrobial effects of nanomaterials on cross-kingdom biofilms.

Name	concentration	biofilm growth/substrate	Treatment time	Microbial number reduction	Biofilm biomass reduction	Cytotoxicity	*In vivo* model	Proposed mechanisms	Reference
CSNP	15%, 30%, 45%	18 h/96-well plate	3h,18h	Reduced (18h, 45%) Other groups no change	Reduced (18h, 45%) Other groups no change	NM	NM	NM	(Ikono et al., 2019)
PG-CSNP	512, 1024 μg/mL	72h/ silicon catheter	24 h	Complete inhibition	Complete inhibition	RAW.267	NM	Destruction of biofilm structure	(Khan et al., 2022)
INOP-CS-CHX	39, 78 μg/mL	24 h/96-well plate	24 h	*Sm*: 3.23, 3.24 log_10 _(39, 78μg/mL) *Ca*: 2.63, 5.91 log_10_(39, 78μg/mL)	53 %(39 μg/mL) 68 % (78 μg/mL)	NM	NM	Inhibition of growth and metabolic activity of biofilms	(Vieira et al., 2019)
NPC-Far	0.5 mg/mL NPC+ 1mg/mL Far	44 h/ hydroxyapatite disks	Five times (0 h, 6 h, 20 h, 26 h, 32 h)	*Sm*: 2 log *Ca*:2 log	Reduced (No specific number)	NM	NM	Inhibition of 3D biofilm organization and disruption of acidification-related activities	(Ito et al., 2022)

*Sm*, *Streptococcus mutans*; *Ca*, *Candida albicans*, CSNP, chitosan nanoparticles; PG, phloroglucinol; INOP, iron oxide magnetic nanoparticles; CS, chitosan; CHX, chlorhexidine; NPC, nanoparticles; Far, farnesol.

Overall, nanomaterials are characterized as a promising therapeutic platform for the treatment of cross-kingdom biofilms. However, two challenges threaten the wide application of nanomaterials for the eradication of biofilms: 1) the possible resistance induced by repeated use of nanomaterials; 2) the potential toxic effect and damage from nanomaterials. The way to reduce the toxicity of nanomaterials is to synthesize nanomaterials by using some biodegradable biomaterials or by self-assembly. There still exists a widening gap between *in vitro* laboratory investigations and clinical practice. The current studies test the antibacterial efficacy through *in vitro* biofilms on polypropylene materials rather than tooth surfaces, which is far from the development of carious biofilms. The associated toxicities, potential side effects to surrounding cells, and lesser biocompatibility lack investigation. Furthermore, the underlying antimicrobial mechanisms and drug resistance possibility of nanoparticles should be revealed in the further work.

## Probiotics

Probiotics refer to live cultures of microorganisms that could influence the composition of microbial communities and confer health benefits on the host when administered in adequate amounts. Probiotics have evolved as an alternative treatment of oral infectious disease owing to their reported beneficial effects in modulating the chronic inflammatory conditions of the gut. As the etiology of oral infection originates from the dysbiosis of microbial communities, the administration of probiotics may contribute to the clearance of pathogens, favor the growth of beneficial species and reduce the biofilm virulence. For instance, the consumption of probiotics in adequate amounts is effective in reducing cariogenic pathogen amounts through competing colonization sites in the mouth and secreting some harmful substance (Sivamaruthi et al., 2020). Moreover, probiotics are participated in the interference of periodontitis via their inhibition of periodontopathogens and modulation of the immune response (Nguyen et al., 2021).

To date, *Lactobacilli* and *Bifidobacterium* are commonly studied probiotics, which produce lactic acid and other substances such as carbon peroxide and bacteriocins. The roles of *Lactobacillus plantarum* (*L. plantarum*) on the inhibition effect of *S. mutans* and *C. albicans* have been investigated. In a rat model, *L. plantarum* CCFM8724 decreased the carriage of *S. mutans* and reduced the caries score in rats (Zhang et al., 2020). The same group extended to investigate the disruption mechanisms of *L. plantarum* CCFM8724 by using metabolomics and transcriptomics analysis (Zhang et al., 2021). They found that some carbohydrates related to biofilm formation were decreased and virulence genes associated with adhesion were downregulated. Moreover, the supernatant of *L. plantarum* 108 could inhibit the biofilm formation of *S. mutans* and *C. albicans*, both in single-species and dual-species (Srivastava et al., 2020). In a recent study, Zeng et al. have assessed the effect of four Lactobacilli strains (*L. plantarum* ATCC 8014, *L. plantarum* ATCC 14917, *Lactobacillus rhamnosus* ATCC 2836, *Lactobacillus salivarius*) on the cross-kingdom biofilm of *S. mutans* and *C. albicans* (Zeng et al., 2022). The data suggested that *L. plantarum* possesses superior inhibition ability on the planktonic growth and biofilm formation of *S. mutans* and *C. albicans*, reduction in EPS matrix production, and microcolonies structure formation. *Streptococcus parasanguinis* is another beneficial commensal that could secrete antimicrobial substances to inhibit the growth of pathogens. The co-culture of a multi-species biofilm model of *S. parasanguinis*, *C. albicans*, and *S. mutans* presented a relative decrease in biofilm biomass compared to co-culture without *S. parasanguinis*, indicating *S. parasanguinis* exert the disruption effect on *C. albicans* and *S. mutans* synergy (Huffines and Scoffield, 2020). Though *S. parasanguinis* reduces the viability of *S. mutans* in a H_2_O_2_ and nitrite-dependent manner, the lack of H_2_O_2_ production in *S. parasanguini*s did not affect the formation of *S. mutans* and *C. albicans* cross-kingdom biofilm. The results revealed that *S. parasanguinis* directly impaired the GTF activity of *S. mutans*, which prevents the GtfB-mediated binding to *C. albicans* mannan and glucan synthesis of *C. albicans*, thereby blocking the synergistic relationship between *C. albicans and S. mutans*.

In summary, probiotics examined using *in vitro* models show a promising method for the elimination of *C. albicans* and *S. mutans* cross-kingdom biofilm. Despite their potential effect on biofilm clearance, probiotics also have some drawbacks that constrain their application as a drug. On the one hand, most probiotics present a low retention rate in the oral cavity. Probiotics incorporated by fast-release formulations cannot ensure enough contact time between probiotic cells and the oral mucosa or dental surface. In addition, probiotics are not exempt from antibiotic resistance. Based on the dynamic condition of the caries biofilm development, more studies should extend the *in vitro* models to diverse and more biologically complex clinical situations. Besides, the effects of probiotics on long-term changes in microbiome composition merit further investigation.

## Antimicrobial peptide

AMP has been considered as an alternative drug to antibiotics due to their several attractive properties, namely the ability to inhibit a broad spectrum of pathogens, the potential to downregulate virulence genes, and the low possibility to induce antimicrobial resistance. They are small-molecule polypeptides that can be divided into natural and synthetic antimicrobial peptides. There are several proposed mechanisms accounting for the direct killing of AMP. The most recognized antimicrobial mode of AMP is that they can insert into membrane bilayers and form transmembrane pores, which could be described by some models like ‘barrel-stave’ pore, ‘carpet’ or ‘toroidal-pore’ mechanism (Batoni et al., 2011; Niu et al., 2021). AMP could accumulate on cell surfaces of microbes, inhibit cell wall formation and induce cell membrane depolarization, leading to microbial death. Besides, AMP can also quickly penetrate microbial cell membranes to attack internal biological macromolecules and interfere with the normal metabolic activities of pathogens (Bechinger and Gorr, 2016).

The human cathelicidin AMP LL-37, has the potential for biofilm elimination, as demonstrated against *S. mutans* biofilm by inhibiting bacterial growth and biofilm formation (Wuersching et al., 2021). Another study has shown that LL-37 binds to the cell wall of *C. albicans*, alters the cell wall integrity, and affects cell adhesion of *C. albicans*, exerting anti-biofilm activity against *C. albicans* (Hsu et al., 2021). A clinical study also revealed the protective role of salivary LL-37 against ECC lesions (Colombo et al., 2016). Other AMPs including L18R, WMR-K, and gH625, have been demonstrated to have antimicrobial capacity on *C. albicans* or *S. mutans* biofilm (Di Fermo et al., 2021; Galdiero et al., 2021; Maione et al., 2021). Simon and co-workers synthesized 75 kinds of cyclic dipeptides and found that cyclic dipeptides containing two aromatic amino acids have antibacterial activity and can effectively inhibit the adhesion of *C. albicans* and *S. mutans* to HA disk (Simon et al., 2019). The antibacterial mechanism may be related to interfering with the expression of quorum-sensing signal molecules. GERM CLEAN is a newly synthesized antibacterial peptide molecule with potent anti-biofilm activity. Xiong et al. have studied its effect on *C. albicans* and *S. mutans* biofilm, their results show that 50% GERM CLEAN can effectively reduce the formation of mixed biofilm, inhibit its acid production and prevent enamel demineralization (Xiong et al., 2021).

The advent of AMP has increased the availability of treatment in *C. albicans* and *S. mutans* cross-kingdom biofilm, however, some challenges still exist in their therapeutic utility. As most experiments were performed *in vitro* without mimicking the oral environment, the long-term stability of AMP in the oral cavity is still questionable. The caries biofilm often results in low pH of the local microenvironment, creating a harsh survival environment for AMP and making them lose biological activity. The antimicrobial and anti-biofilm performance of AMP may be dramatically affected. Several investigations have tried to use different chemical modification methods to extend the half-life of AMP, including the substitution of standard amino acids with non-natural amino acids, peptide cyclization or terminal modification via amidation, alkylation or acetylation. AMPs are expensive to manufacture under the scale of industrial production since their extraction, isolation, and purification processes are complex. Moreover, though AMP has a lower chance of inducing antimicrobial resistance than antibiotics, some bacteria harbor sensors that could activate AMP resistance mechanisms. Therefore, some aspects of AMP need to be explored further, such as the improvement of long-term stability, the controlled maintenance of bioactivity, and bacterial resistance.

## Antimicrobial photodynamic therapy

aPDT is a process in which a non-toxic substance called photosensitizer interacts with the light source in the presence of oxygen. After receiving the appropriate wavelength of light illumination, the photosensitizer would undergo intersystem crossing to transform into a much longer-lived triplet photosensitizer. Then the triplet photosensitizer can interact with molecular oxygen via electron or energy transfer to produce reactive oxygen species (ROS) (Hu et al., 2018). These ROS are highly reactive and could target various cellular components leading to microbial death. Based on previous publications regarding antimicrobial mechanism of aPDT, it is proposed that aPDT undergoes two steps to fight against virulent biofilms. Firstly, the photosensitizer should penetrate the biofilm matrix and then bind to microbial cell surface or enter into the cellular cytoplasm. Once the photosensitizer is localized, the generated ROS could induce a series of attacks on adjacent molecules, including biofilms matrix components, cell wall or cell membrane, and inside the cells.

Due to this multi-target action of aPDT, it is reasonable to assume that aPDT can inactivate bacteria regardless of the level or mechanism of bacterial resistance. More importantly, aPDT has less opportunity to induce antimicrobial resistance when compared to antibiotics. Accumulating evidence has demonstrated the anti-planktonic and anti-biofilm effects of aPDT on gram-positive bacteria, gram-negative bacteria, and fungus (Pinto et al., 2018; Cai et al., 2019). Therefore, aPDT has been proposed to be a promising alternative in the treatment of oral infectious diseases. Gong et al. have investigated the effect of the combination of eosin and light-emitting diode on a multi-species biofilm including *S. mutans, C. albicans* and *Lactobacillus casei* (*L. casei*) (Gong et al., 2019). The CFU data showed the amount of *S. mutans*, *C. albicans* and *L. casei* was reduced by 96.64%, 97.59% and 83.33% respectively after aPDT treatment. Other articles also have investigated the antimicrobial effects of photosensitizers such as methylene blue and curcumin against *S. mutans* and *C. albicans* dual-species biofilm after irradiation (Pereira et al., 2010; Quishida et al., 2016). These results are hardly comparable due to multiple factors including bacteria species, photosensitizers, and aPDT parameters, but they all revealed that aPDT treatment alone is not enough to eradicate the cross-kingdom biofilm. Some photosensitizers are highly lipophilic and tend to aggregate in the aqueous environment, leading to the loss of photosensitizing activity and limited therapeutic index. Therefore, some endeavors have been made to overcome the situations and improve the efficiency of aPDT. As curcumin is water-insoluble, some studies have encapsulated curcumin in nanoformulations for increasing water solubility and antimicrobial effectiveness. The encapsulation of curcumin into Pluronics®-127 nanoparticles exhibited the antimicrobial effect against the *S. mutans* and *C. albicans* biofilms with more than 3 log steps of reduction (Dantas Lopes Dos Santos et al., 2021), which has achieved the bactericide effect stated by the USA Food and Drug Administration’s Tentative Final Monograph. Though the enhancement of antimicrobial effectiveness was not observed compared to unloaded curcumin, the curcumin loaded with Pluronics®-127 exhibited adequate polydispersity index, stability, lower photodegradation, and autoaggregation compared to unloadedcurcumin. Another study has investigated the photodynamic action of chloroaluminium phthalocyanine in chitosan nanoparticles on cross-kingdom biofilm (Trigo-Gutierrez et al., 2018). Interestingly, the result showed that the encapsulated chloroaluminium phthalocyanine reduced the viable counts and metabolic activity of multi-species biofilm, whose antimicrobial effect is superior to 0.2% CHX.

The antimicrobial effectiveness of aPDT is dependent on multiple factors including the characteristics of photosensitizers, oxygen concentration, light dose and so on. aPDT is currently only proposed as an adjunct disinfection method in dental applications, mainly due to EPS matrix and efflux pumps of bacteria that hinder the binding capacity of photosensitizers and thus affect the antibacterial effect. Moreover, the generated ROS has a short lifespan and diffusion length in the biological environment. The specific structure and hypoxic environment of dentin tubule also confine the effect of aPDT. Further studies should concentrate on developing new photosensitizers with good hydrophilic properties and high reactive oxygen species yields or improving the delivery efficiency of photosensitizers in order to achieve high antimicrobial efficacy.

## Combination therapy

The complex structure of cross-kingdom biofilm and its elevated virulence pose major challenges to currently available antimicrobials (Ponde et al., 2021). As some antimicrobials have difficulties penetrating the dense matrix, the concentration of drug within the biofilm is too low to kill pathogens. Concerted efforts have been made to develop new antimicrobials, nonetheless, no new classes of antibiotics or their alternatives have been clinically approved in the last three decades. Given the recognition of the difficulties in drug innovation, combining the use of two or more antimicrobial agents may be a feasible and cost-effective strategy. Combination therapy has recently become optional for treating multidrug-resistant pathogens or complex cross-kingdom since it often leads to a more desirable outcome (Tyers and Wright, 2019; Zhang et al., 2022).

For example, aPDT has been paired with natural compounds, antibiotics, and chelating agents to enhance antimicrobial efficacy against refractory biofilms. These combinations possess many benefits, including less induced antimicrobial resistance, the reduction of side effects, the provision of alternative action pathways, and the improvement of the antimicrobial efficacy. Our previous work has compared the effects of toluidine blue O (TBO)-mediated aPDT in combination with H_2_O_2_ or not on *S. mutans* and *C. albicans* dual-species biofilm formed on polymethyl methacrylate disk and demonstrated that H_2_O_2_ administration followed by aPDT treatment displayed the highest capacity in disinfecting biofilm compared to either treatment alone (Li et al., 2021). Additionally, pretreatment with H_2_O_2_ could degrade EPS of biofilm and increase the outer membrane permeability of microbial cells, which are beneficial to the absorption of photosensitizers by microbial cells and further improves the effectiveness of aPDT treatment. The combination of antibiotics or antifungal drugs with chemotherapy is a feasible approach to treat difficult-to-treat infections and improve the therapeutic outcome. Kim et al. cultured *S. mutans* and *C. albicans* on HA disks for 43 h to form the cross-kingdom biofilms and investigated the combination effects of povidone iodine (PI) and fluconazole on biofilm (Kim et al., 2018). Interestingly, the combination exhibited higher antimicrobial efficacy than using PI or fluconazole and resulted in the complete elimination of *C. albicans* from HA disks. It has been documented that PI enhanced the uptake of fluconazole in *C. albicans* by disrupting the assembly of EPS matrix shield through inhibition of α-glucan synthesis by *S. mutans* bound on the *C. albicans* surface. Another example is the combination of Cis-2-decendnoic acid (C2DA) with CHX on dual-species biofilm (Rahmani-Badi et al., 2015). C2DA is a type of fatty acid secreted by *Pseudomonas aeruginosa* and could exert an anti-biofilm effect at nano-molar ranges. It was noteworthy that 100 nM C2DA and 0.06% CHX lead to the complete elimination of cross-kingdom biofilms, both of which decrease the dose required to achieve the same eradication effect. Although the cooperative actions of H_2_O_2_ or CHX with some antimicrobial strategies have been proven previously, the side effects of high-concentration of H_2_O_2_ or CHX application should not be ignored. These side effects include taste alteration, tooth staining, burning sensation, and toxicity to oral mucosal cells. Therefore, it is not recommended to use H_2_O_2_ and CHX on children every day. However, there is one example where the combination of pH-responsive nanoparticles with three different drugs (tt-farnesol, myricetin, and compound 1771) showed limited efficiency against cross-kingdom biofilms (Roncari Rocha et al., 2022). Among them, tt-farnesol could target the cell membrane of microorganisms to exert an antibacterial effect; myricetin inhibits F-ATPase activity and suppresses the gene expression related to Gtfs; the compound 1771 inhibits the synthesis of lipoteichoic acid in Gram-positive bacteria to hinder the early coaggregation. The combination of three drugs had no efficacy on established *S. mutans* and *C. albicans* dual-species biofilms and only reduced the early colonization of microorganisms on the surface.

Antimicrobials reported to date do not meet the criteria of a “magic bullet”, it is likely that we enter into the “post-antibiotic” era. The intricate interaction, spatial organization, and altogether behaviors of cross-kingdom biofilms reinforce the need to switch from monotherapy to combination therapy. From the studies we discussed, we could find the combination of two or more antimicrobials provides multiple sites of action to complement monotherapy, which is beneficial to the treatment of cross-kingdom biofilms formed by different structured microorganisms. Besides, the combination leads to a decrease in the antimicrobial concentration used and avoids some side effects. However, there are numerous questions left unanswered regarding standardized combination therapy. Firstly, the synergistic effect is not concluded from the enhancement of antimicrobial efficacy after applying combined antimicrobials. The synergistic interaction among antimicrobials should be evaluated through the assessment of fractional inhibitory concentration index. Secondly, the variables of combination therapy should be considered and reported in order to give a full comparison among studies. The treatment order, antimicrobial dosage and treatment frequency are linked to the final outcome. Additionally, several disadvantages of combination therapy should be considered. Not all combinations of antimicrobial methods could ultimately increase antimicrobial effectiveness. And, some combinations of antimicrobial strategies are only effective for certain species and cannot achieve broad-spectrum coverage. Under such circumstances, unnecessary antibiotic exposure that fuels resistance in the patient and the healthcare setting may occur.

## Conclusion and future perspectives

Caries remain a tricky challenge worldwide, threatening individuals in developing and developed countries. The *S. mutans* and *C. albicans* cross-kingdom biofilm has been demonstrated to orchestrate the development and progression of caries. The specific features of the cross-kingdom biofilm, especially their mutual metabolic interaction, stability to mechanical forces, ability to produce EPS matrix, and resistance to current antimicrobials, make them an important clinical issue. However, many understandings regarding cross-kingdom biofilm are still uncovered such as resistance regulatory mechanisms and persistent cell development. There are also some new molecular targets needed to explore. The cognitions of the formation and regulation mechanisms of cross-kingdom biofilm, together with the validation in an *in vivo* model, are essential steps against cross-kingdom biofilm in caries treatment.

Over the past decade, there has been movement regarding innovative strategies against the cross-kingdom biofilm. The non-antibiotic therapeutic approaches, especially those natural extracts and nanomaterials, have shown significant potential in inactivating microorganisms and eliminating biofilm. Of the antimicrobials or strategies reviewed here, an efficient method to treat the cross-kingdom biofilm may be exploiting combinatory therapy. Combination therapy has shown advantages over standalone treatments such as reducing the concentration of the drug, enhancing selectivity and effectivity, and reducing the possibility of resistance. There are considerable uncertainties about these newly emerged treatments, which require further work to be done before implementing them in the clinic.

Herein, some considerations should be taken into account when developing new drugs or introducing new approaches for the elimination of *S. mutans* and *C. albicans* cross-kingdom biofilm. First, the toxicity profile of new antimicrobial agents should be concerned and thoroughly investigated before full-scale translation to the clinic. The main clearance route of oral topical treatment is via ingestion, which may lead to systemic circulation and tissue distribution. Several studies have reported systemic side effects due to the use of metal or metal-oxide-based nanoparticles (van der Zande et al., 2012; Lee et al., 2018). It is better to perform short-term and long-term evaluations to ensure the biosafety of new antimicrobial agents. Second, a concern of any new drug or strategy is the development of antimicrobial resistance, which should be explored in detail. Though the actions of some antimicrobial approaches like nanoparticles or aPDT are a combination of mechanisms, repeated exposure to any specific antimicrobial approach may result in variations of the targeted pathogens. Third, maintaining the balance of innate oral flora must be established during the use of a new drug or strategy. Indiscriminate use of antibiotics may lead to oral ecosystem dysbiosis again and aggravate the disease. The clinical goal of a new drug or strategy should not only aim to reduce pathogenic microbes but also to consolidate the harmonious balance of oral flora. Fourth, topical antibacterial agents are not maintained at the necessary level for a long duration due to the rapid clearance of saliva. To circumvent these problems, drug delivery systems like liposomes and micro particles are designed to target hydroxyapatite or bind with pellicle protein in order to enhance the bioavailability and retention of new compounds at dental surfaces and periodontal pockets. In addition, some studies have incorporated pH-responsive moieties into nanoparticles to ensure drug delivery in acidic pH within the cariogenic microenvironment. Fifth, patient compliance and motivation are essential in the prevention and treatment of caries. If child compliance is poor or the antimicrobial is discontinued before the elimination of pathogenic microbes, recurrent caries may occur because remaining viable microorganisms will have the ability to grow and replicate. It therefore makes sense to develop new antimicrobial agents that do not require patient education for strict compliance. For instance, replacement therapy like probiotics may ensure lifelong protection after a single application without a need for long-term compliance. Besides, the other feasible method to improve patient compliance, it seems reasonable to move from local administrations by professionals to consumer products and home-care procedures, such as antimicrobial components incorporated into dentifrices and chewing gums. Sixth, economic consideration of new antibacterial compounds or strategies, in terms of production cost, is another important aspect in developing new drugs. It remains unclear whether the cost-benefit ratio of the new strategy will overcome traditional treatment regimens such as mechanical clearance, antiseptic use, or topical fluoride applications. The cost-effectiveness of the new strategy should be considered alongside the antimicrobial efficacy. Seventh, in addition to microbiological analysis, new antimicrobial drugs or approaches need further investigation on their practicality and feasibility. For instance, if the new drug is developed for daily use, examining the interaction of the new drug with currently available products like fluoride toothpaste may be required for compatible evaluation. Eighth, dental caries are caused by multi-species biofilms attaching to the dental surface or restorative materials. The artificial biofilm models range from monoculture to multi-species biofilm models. It is important to consider the shortcomings of biofilm models under *in vitro* and *ex vivo* conditions from clinical aspects. Single-species biofilm, predominantly *S. mutans*, is easy to grow and replicate in most experimental conditions. Although mono-species biofilm possesses high cariogenic potential, it cannot stimulate complex interspecies interactions related to high virulence and increased antimicrobial tolerance. Multi-species biofilm or naturally isolated biofilm are candidates for future studies to examine antimicrobial agents. In addition, substrates for biofilm culture are also an essential part of determining the effectiveness of antimicrobial agents. Synthetic surfaces like glass, polystyrene, or polydimethylsiloxane cannot reproduce the microanatomy of dentine and the interaction between the tooth surface and antimicrobial agent. It has been suggested that human dentin is a good choice for caries biofilm growth. Moreover, biofilm growth conditions, such as static or dynamic, will influence biofilm biomass, which in turn affects the evaluation of new antibacterial strategies against biofilms. Other factors like biofilm age, culture medium, and substrate size also influence the assessment of antimicrobial methods. However, there is no standardized protocol for biofilm model construction for antimicrobial assessment so far. Standardized biofilm models should be made to represent a similar oral environment, which will help researchers find potent therapeutic strategies.Therefore, future work is recommended to evaluate the overall aspects of the newly antimicrobials or strategies, which shall help in introducing effective approaches into clinical practice.

Apart from those strategies we reviewed here, we found that the physiological cell-to-cell interactions in *S. mutans* and *C. albicans* biofilm can indeed serve as another avenue worth exploring as the potential treatment target. Gtfs and glucans play a role in *S. mutans* and *C. albicans* interaction, which can be considered the primary targets for developing eradication strategies against cross-kingdom biofilms. Targeting Gtfs or glucans by hampering their activity and consequently inhibiting the production of EPS would impair the co-adhesion between *S. mutans* and *C. albicans*, which is an appealing strategy for cross-kingdom biofilm prevention. Another plausible strategy to treat *S. mutans* and *C. albicans* cross-kingdom biofilm is to inhibit the production of their adhesins, which are crucial to the formation and development of cross-kingdom biofilm. While some inhibitors of intercellular signaling of *S. mutans* or *C. albicans* biofilm have been identified in previous studies, little progress has been made in these inhibitors targeting *S. mutans* and *C. albicans* dual-species biofilm. Targeting metabolic pathway regulators such as farnesol, CSP, or XIP is anticipated to interfere with the cell-to-cell metabolic interaction and thus play a potential role in the treatment of dual-species biofilms.

## Author contributions

YL, MW and XH contributed to the conception and design of the study. YL, SH and JD performed the data searching and wrote sections of the manuscript. YL wrote the first draft of the manuscript. XH performed the final corrections. All authors contributed to the manuscript revision and read and approved the submitted version.
